# The social amplification of risk framework: New perspectives

**DOI:** 10.1111/risa.13926

**Published:** 2022-07-20

**Authors:** Roger E. Kasperson, Thomas Webler, Bonnie Ram, Jeannette Sutton

**Affiliations:** ^1^ Geography Department and George Perkins Marsh Institute Clark University Worcester Massachusetts USA; ^2^ Social and Environmental Research Institute Shelburne Massachusetts USA; ^3^ Center for Research in Wind University of Delaware and Ram Power Consultancy Washington District of Columbia USA; ^4^ College of Emergency Preparedness Homeland Security and Cybersecurity, University at Albany, SUNY

## Abstract

Several decades have elapsed since the introduction in 1988 of the social amplification of risk framework (SARF) by researchers from Clark University and Decision Research. SARF was offered as an umbrella under which social, psychological, and cultural theories of risk could be integrated and thereby supplement technical risk analyses. Some critics suggest that SARF cannot be tested thus, the framework is useful, at most, as a *post hoc* analysis of some kinds of risks. Others counter that predictability is not required for a framework to be useful and that SARF is an effective tool in organizing data related to public perceptions, values, and behaviors. It can also be used to design more effective risk communication and public engagement strategies. SARF also suggests how to conceptually view the dynamics of social media channels, despite the fact that SARF was developed before the explosion of global digital platforms. The papers in this special issue consider developments, refinements, critiques, contributions, extensions of the approach to new risk issues, as well as the findings and hypotheses that have grown out of what is now close to three decades of empirical research. This introductory paper provides background on SARF, presents a literature review since 2003, introduces the contributions to this issue, and highlights several areas for future research.

## 1 THE ANALYSIS OF RISK: BACKGROUND

In the risk analysis field, hazards and risks are still primarily defined in a technological sense (National Research Council, 1983) although hazards are not only threats to people, but what they value as well (Kates et al., 1985). This was also made clear in the *Understanding Risk* report (NRC, 1996). Still, a technical definition of risk that focuses on loss of life and an analytic approach limited to a sequence of natural and technical events leading to loss pervades the discipline. There is limited social science theory to broaden these approaches, although several have been suggested such as:

- Psychometric theory and affect heuristics: how individuals make judgments about risk and hazards and how reliance on our emotions relates to decision making, respectively (Slovic et al., 1986);
- Media and communication theories: how we communicate about risk and uncertainty (Arvai & Rivers, 2014; NRC1989);
- Cultural theory: how cultural worldviews preference risk perceptions (Johnson & Swedlow, 2021);
- Organizational theory and political economy: how social forces enter into public policy and decision making (Freudenburg & Pastor, 1992; Majone, 1989; Rip, 1986).

But integrated analysis, especially in the risk community, is still lacking. Fischhoff (1995) made it clear that we need to take account of technical risk, but that is not enough. Although social science work—particularly psychometric analysis—has been widely circulated, technical analysis of risk still dominates and there is an underappreciation of the social, political, and cultural dynamics that influence risk characterization and risk management. This often leads policymakers and experts to adopt the belief that the solution is not to broaden their understanding of risk, but to teach publics and stakeholders to think like they do. Activities and reports by federal and state governments, and even the National Academy of Science (National Academy of Sciences, 2017), reveals that this so‐called “knowledge deficit model” is still inappropriately influential. At stake is the issue of whose perceptions, values, and beliefs matter? This is not to say, as Fischhoff points out, that the technology is not a source of serious risk. It is. But technological risk assessment is only part of the risk analysis challenge. Who determines the definitions of risk and hazard and how those definitions are used by various parties are also critical.

## 2 THE SOCIAL AMPLIFICATION OF RISK FRAMEWORK

SARF is a conceptual framework and not a theory. Its foundations are developed in six principal publications (Burns et al., 1993; Kasperson, 1992; Kasperson et al., 1988; Kasperson, & Kasperson, 1996; Pidgeon et al., 2003; Renn, 1991). Its goal was to assess the technical issues of risk and their interaction with the psychological, sociological, and cultural perspectives of risk perception and risk‐related behavior. The main premise was that these issues of human behavior cannot be considered epi‐phenomena of technical risk analysis because hazards interact in multiple social ways with technical issues that may result in amplification or attenuation not only of public perceptions and responses, but also of the risk itself (see Larson et al., 2022, this issue). Thus, SARF was not only supposed to bring in social science research, it also was supposed to connect in an integrative manner the social science with technical analyses.

The idea for this framework arose out of an attempt to overcome the fragmented nature of risk perception and risk communication research by developing an integrative framework capable of accounting for findings from a wide range of studies, including media research; psychometric and cultural schools of risk perception research; and studies of organizational and societal responses to risk. The framework also serves, more narrowly, to describe the various dynamic social processes underlying risk perception and response. In particular, those processes by which certain hazards and events that experts assess as relatively low in risk can become a particular focus of concern and sociopolitical activity within a society (risk amplification), whereas other hazards that experts judge to be more serious receive comparatively less attention from society (risk attenuation). Attenuation can stem from threats to deeply held values and the conscious attempts to avoid systematic (or slow) thinking. Examples of significant hazards subject to attenuation might include naturally occurring radon gas, automobile accidents, smoking, and electronic cigarettes. Risk amplification typically occurs at two stages: in the transfer of information about risk and in social responses. Signals about risk are both transmitted and processed by individuals and social entities, which are called “amplification stations” in the literature. The individual might be a scientist, for example, who communicates the risk assessment. A social entity might be a news media, a cultural group, or an interpersonal network (Hill, 2001). The perceived amplified risk may lead to behavioral responses that can result in secondary impacts or “ripples.” Ripples include phenomena such as stigma–negative connotations associated with a community hosting a radioactive waste management facility, for example.

Social amplification may qualitatively and quantitatively increase not only signals about the risk but also perceptions of risk, behaviors related to the risk, as well as the risk itself and its consequences (Funtowicz & Ravetz, 1990; Kasperson et al., 1988). For this reason, social amplification of risk should be included in analyses of public and regulatory reactions to risk events. The key amplification stages are posited as:

- filtering signals (only a fraction of all incoming information is actually processed);
- decoding and reframing the signals;
- processing risk information (e.g., drawing inferences);
- attaching social values to information as a basis for drawing implications for management and policy; and
- behavioral change of individuals and institutions.

The societal information system may amplify hazard events in two major ways:

- by intensifying or weakening signals that are part of the information that individuals and social groups receive about the hazard or
- by filtering the multitude of signals with respect to the attributes of the hazard and their importance (Renn, 1991; Renn et al., 1992).

Signals arise through direct personal experience with a risk object or through the receipt of information about the hazard from the information ecosystem and/or personal networks. These signals are processed by social, as well as individual, amplification stations that include:

- the scientist who conducts and communicates the technical assessment of risk;
- risk management institutions;
- social media channels and platforms and the traditional print and broadcast news media;
- activist social organizations;
- opinion leaders within social groups (e.g., influencers);
- personal networks of peer groups;
- public agencies.

Social amplification stations generate and transmit information via communication channels (traditional media, social media, and direct conversations). In addition, each recipient also engages in amplification or attenuation processes, thereby acting as an amplification station for risk‐related presentations.

**FIGURE 1:**
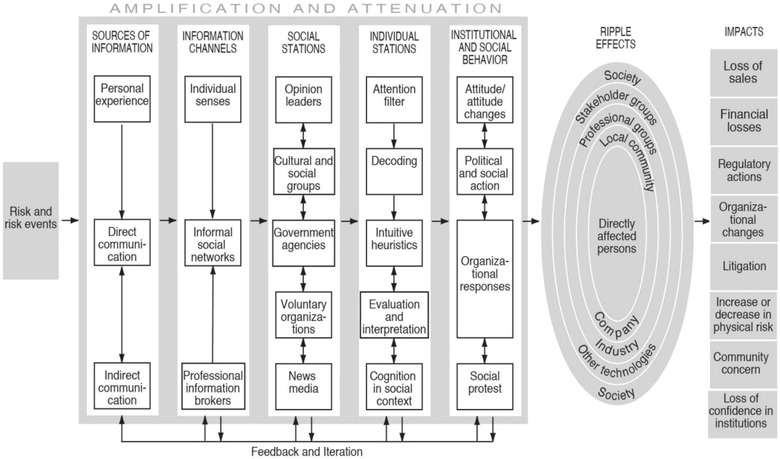
Social amplification of risk framework. *Source*: Kasperson and Kasperson (1996)

Originally SARF's authors imagined ripples in a pond as a way to think about how impacts associated with the social amplification of risk spread outward or stay inward (Pidgeon et al., 2003), but this was prior to global digital communication platforms. Time and space are both important in such rippling topics (Figure 1).

Now with ubiquitous social media channels, these ripples have the potential to fan out in minutes to global communication networks. The effects that are involved are diverse and diffuse and include such impacts as:

- losses in local business sales, lower residential property values, and lower levels of economic activity;
- political and social pressure (e.g., political demands, changes in the political climate and culture);
- changes in the nature of the risk (e.g., feedback mechanisms that heighten or lower the risk);
- changes in training, education, or required qualifications for operations and emergency response personnel; changes in risk monitoring and regulation;
- higher liability and insurance costs;
- repercussions on other technologies 1 (e.g., lower levels of public acceptance) and on social institutions (e.g., erosion of public trust); and even
- social disorder (e.g., protests, riots, sabotage, terrorism).

All of these are, of course, examples of “risk ripples” and are part of the consequences of risk. One of the strengths of SARF is anticipating the possibility of multiple feedback loops and that risk management could be affected by historical perceptions of risks (see Cox et al., 2022, this issue). Another strength of the framework is that it accommodates many types of hazards, for example, in this special issue SARF is applied to vaccines, fracking, and opioids.

**FIGURE 2:**
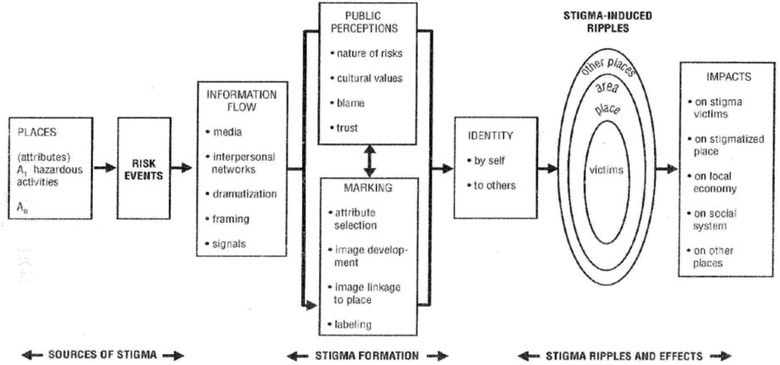
Risk Amplification and stigmatization. *Source*: Kasperson et al. (2001)

An extension of the psychometric paradigm led to the definition of stigma (Figure 2). Stigma refers to the negative imagery associated with undesirable social groups or individuals (Flynn et. al, 2001; Goffman, 1963). But environments with heavy pollution (Love Canal, Times Beach, Fukushima, Nevada Test Site, etc.), hazardous technology (hazardous waste incinerators, chemical weapons disassembly factories), and other controversial hazards such as radioactive waste disposal, or even relatively benign technologies (e.g., solar and wind sites) may also come to be associated with negative images (Slovic et al., 1994). Since the typical response to stigmatized persons or environments is avoidance, it is reasonable to assume that risk‐induced stigma may have significant social, economic, and policy consequences (Flynn et al., 2001).

The creation of SARF stemmed from technologies and incidents that were considered “high hazards” (e.g., chemical explosions and nuclear accidents). Researchers are now exploring how SARF can support assessments around technologies with the potential for significant benefits along with uncertain hazards (see, e.g., Ram & Webler, 2022 in this special issue examining an offshore wind power case).

## 3 LITERATURE REVIEW

Fifteen years after SARF was created, progress in developing and employing the framework was reviewed (Pidgeon et al., 2003). It found that SARF had gained considerable traction, despite meeting with criticisms. We identified over 50 research articles on SARF published since 2003 and found it continues to be a popular framework to help interpret the social dimensions of risk. The original article on SARF is the seventh most cited article in *Risk Analysis*, with 40 citations in 2021 alone. In particular, considerable attention is now being paid to social media and the processes by which messages alter risk perceptions. Clearly, most of the literature on SARF concerns amplification, although attenuation has long been recognized as important and as prevalent. A recent article focused on strategically including uncertainty information in risk characterization to intentionally inject more amplification into the process and thus counter the effects of risk attenuation (Fjaeran & Aven, 2021).

A foundational critique of SARF raised by Rayner (1988) and Rip (1988) has been addressed by Busby et al., Duckett, and others. Rayner claimed that SARF gives preference to a “correct” or “objective” expert risk characterization that is then “distorted” through communicative and societal processes. Busby et al. suggest a reorientation to a more relativistic approach that draws attention away from questions of epistemological hegemony to questions about how actors can hold views about risks that others, some more numerous, knowledgeable, or powerful, contest and how “a society of actors construct each other's risk responses” (Busby & Duckett, 2012, pp. 1051; Busby et al., 2009; Busby & Onggo, 2013; Duckett & Busby, 2013). Such a perspective has been taken up by others. For instance, Carper (2019) studied vaccination decisions and found an informational channel (specifically, stories shared among friends) had the largest impact on risk behavior.

A great deal of the literature citing SARF since 2003 examines media coverage as indicators or influencers of public risk perception, although more effort is put into measuring media coverage than is put into clarifying the process by which media coverage influences risk perceptions. Since SARF is not measurable, one could argue that “diffusion” and “retransmission” is amplification (Sutton et al., 2015; Vos et al., 2018). It has been well‐documented that SARF originated in a period preceding social media, which offers unique challenges to the framework. An article by Chung (2011) is often cited as the first to argue social media demands reconsideration of the structure of SARF. In the decade following Chung's article, a modest amount of progress has been made. Comrie et al. (2019) used system dynamics modeling to operationalize how social media contributes to risk amplification about acute health risks. They found that failure of government health offices to participate in social media leads to more misinformation, which can result in lower trust (as Frewer, 2003 also explained) and less institutional control over risk management. Importantly, they also examined the challenges that public health agencies face in participating effectively in social media. Hopfer et al. (2021) illustrated how social media can both amplify and attenuate risk at the same time but among different audiences. Moussaïd et al. (2015) offered a particularly intriguing explanation of how social media distorts risk characterizations using the theory of diffusion chains. They reported that messages become shorter, less accurate, and dissimilar as they are passed along a 10‐subject chain.

Fellanor et al. (2018, 2020) suggested that social media be considered an amplification station, and that it blurs the lines between journalist and news consumer. One of the qualities of social media as an amplification station that Fellanor et al. highlight is that risk amplification becomes intertwined with one's motive of self‐promotion. People attempt to piggyback their personal interests onto the ongoing risk debate. However, it remains unclear if this is unique to social media. The behavior of social media users to share and seek information is a growing area of study. A recent study found that Twitter users were less inclined to share risk information if they were concerned about the presence of misinformation (Zhang & Cosma, 2022). Another compared traditional and social media's role in shaping risk perceptions of forest fire haze and dengue fever. They found social media was much more effective, possibly because it was easier to share images that documented the haze (Ng et al., 2018). Wirz et al. (2018) investigated how messages about blame and stigma varied across social media platforms in Spanish, Portuguese, and English. Clearly, more research into social media and SARF is needed (see Section 5.1 below).

Another focus of recent scholarship into SARF has centered on how different types of messages can activate cognitive and emotive responses, promoting certain risk perceptions. For example, Crespi and Taibi (2020) looked at how news media in Germany amplified tourists’ perceptions of earthquake risks in Italy when content emphasized uncertainty and dramatic consequences. Chong and Choy (2018) did something similar, but added social media channels. They found that Facebook was the most effective channel that stimulated emotional responses that amplified risk. Popovic et al. (2020) manipulated subjects’ stress and then asked them to share information about a hypothetical risk. They found that people who were stressed reported lower risk concerns and also attenuated risk via social media.

This synopsis of SARF‐related research published since 2003 illustrates that the framework retains broad appeal as a scaffold to gather and organize data about cultural, societal, and psychological forces that shape risk perceptions and behaviors (See Appendix for the list of articles reviewed).

## 4 HIGHLIGHTS OF THIS SPECIAL ISSUE

The call has long been out in the risk community for more integrated and complete characterization of risks. The contributions of this special issue respond to this call and demonstrate how SARF continues to open up new issues and approaches to risk analysis. A testament to SARF is the diversity and range of topics that are part of this special issue, including: social trust, the opioid crisis in the United States, vaccine hesitancy, offshore wind power, coastal vulnerabilities, systemic risks, and negative emissions technology and fracking in the United Kingdom.

Issues of social trust are clearly important components of the dynamics of social amplification (Earle & Cvetkovich, 1993). We know that distrust in experts can heighten or reduce risk perceptions, intensify or attenuate public reactions to risk signals, contribute to the perceived acceptability or unacceptability of risk, and stimulate political activism to reduce or ignore risk (Slovic, 1993). A host of questions surrounds the interpretation of trust and its effects. There are many types of trust. The processes that create and destroy trust are not well understood. Trust (or distrust) exists at multiple levels of the political system, complex attribution issues prevail, and policy responses and their effectiveness are opaque. From a social amplification perspective, trust is highly interrelated with other components and mechanisms in what we think of as “amplification dynamics.” Understanding how trust is shaped, altered, lost, or rebuilt in the processing of risk by social and individual stations of risk is a priority need in SARF research. Bearth and Siegrist (2022, this issue) contribute here by reviewing the literature on the role of trust in different amplification stations. They then use Fukushima as a case study to explore the possibility that trust in nuclear power operators was compromised by perceived competence to manage nuclear power stations. Consequences may have rippled out to Germany and played a role in their decision to abandon nuclear power by the end of 2022. They suggest that the presumed “death spiral” of trust is empirically unjustified and risk communication suffers if one starts with a defensive stance.

Cantor et al. (2022, this issue) investigate the utility of SARF to explain both attenuation and amplification of risks in the context of the opioid crisis. They draw upon Ortwin Renn's suggestion that functional resonance and tragedy of the commons both help explain amplification. Examining various “signals” related to opioid risks, they found that SARF's focus on the treatment of risk signals helped to understand muted public responses to the increasing levels of the risk signals before 2011, which allowed the opioid crisis to expand and become a national health emergency. Post 2011, they found SARF highlights information regarding certain risk perception triggers, media attention, and a growing public search for accountability. However, what SARF could not do was to appraise the appropriateness of societal responses to opioid risks. The value of SARF, they found, was as an organizational aid to study historical information rather than as a predictive tool for determining inappropriate risk management responses. The paper also found that economic incentives and opportunities are not well emphasized in the attenuation factors and analysis of the role of such economic signals is better addressed directly by socioeconomic theory and empirical study.

Larson et al. (2022, this issue) explore the implications of the SARF for the hazard identified as “vaccine hesitancy.” They show that vaccine hesitancy, a refusal of or resistance to vaccination, fits squarely in SARF. Communication about vaccines and their real and imagined risks can affect perceptions that strongly influence behavior to accept or reject vaccination. Increased rejection can significantly increase the technical risk of getting sick, both for the individuals themselves and the population around them. They also show that altered perceptions and behaviors can open up many further possibilities for ripple effects. Using data collected over the past decade by the Vaccine Confidence Project (based at the London School of Hygiene and Tropical Medicine), they describe several recent examples of social amplification and hesitancy starting with the notorious false association of autism with the MMR (measles, mumps, and rubella) vaccine complex. The separate examples illustrate different aspects of risk amplification processes. Larson et al. (2022) go on to consider some aspects of current experience with COVID‐19 vaccines; SARF, suitably adjusted to account for the ubiquity and power of social media and the systemic nature of the vaccine hesitancy hazard, can shed considerable light on that experience. They conclude that an adjusted SARF serves well to organize descriptions and analysis of vaccine hesitancy. They also assert that the SARF shows promise for guiding practical efforts to ameliorate the occurrence and impact of vaccine hesitancy. Moreover, the authors of this editorial believe that the COVID pandemic will hold new insights and lessons about the theories of risk communication meeting practice in an emergency. It is likely that SARF was useful in pointing out to the medical communities what some of the social processes they were witnessing really meant.

In their analysis of controversies about offshore wind in the United States, Ram and Webler (2022, this issue) make two contributions. First, they point out that few studies have made the point that benefits as well as risks can be amplified or attenuated in risk discourses (see, for example, Roberts, 2019). In the past, SARF has tended to focus entirely on risks with negative consequences. Second, they clarify an important but largely neglected element in SARF: the four informational mechanisms. Early and seminal publications of SARF introduced four mechanisms—volume of information, extent to which factual information is disputed, dramatization, and symbolic connotations—and suggested these were the means by which signals about risks were amplified or attenuated. These were, until now, never given deeper consideration. Drawing on a wealth of qualitative data from two case studies of offshore wind in the mid‐Atlantic US coast and guided by an extensive literature review, they reveal the strategies parties use to influence public risk perceptions and siting decisions. The paper concludes that SARF is useful for organizing qualitative information and sharpening insights on participatory risk governance and the nuances of public responses to a relatively new low carbon technology.

SARF has identified a number of groups and social roles that participate in the amplification or attenuation of risk, but rarely has focused on government actors. Dow and Tuler (2022, this issue) remedy this by pointing to the unique roles played by climate resilience officers in large American cities. In particular, they examine how these individuals adopt either amplifying or attenuating messaging as they strive to reach audiences driven by panic or denial. Resilience officers have a responsibility to help people take mitigative actions, thus need to maintain a focus on how their risk communication efforts inspire or discourage action. They conclude by pointing to several future research directions for SARF. First, by building on agenda‐setting and policy‐framing research to understand political forces at play. Second, they propose using arena theory to examine resilience officers’ strategic decision making about amplifying or attenuating risk messages. Third, they suggest the idea of “risk work” could further inform strategic decisions of resilience officers. Fourth, they recognize value in a deeper understanding of what influences people's interpretation of risk signals.

When it was first proposed, SARF focused on technological risks associated with nuclear power, chemicals, carcinogens, and pollutants. Over the decades, the literature has grown to consider many other types of risk. Schweizer et al. (2022) continue to push this envelope by examining SARF in the context of systemic risk (this issue). In particular, they explore why systemic risks seem to be generally attenuated in public perception (although they can also be amplified). They show how SARF is useful and suggest ways to strengthen the framework by identifying practical tools for assessing the significance of perceptions of systemic risk. They conclude that a number of attributes of systemic risk seem to explain the attenuation including; psychological distance, their nondeterministic and ambiguous nature, and lack of trust in the scientific assessments of systemic risk. Finally, they report that misrepresentation of systemic risk is easily reinforced by digital communication tools which bolster echo chambers in public discourse. Consequently, knowledge camps become polarized and differentiated approaches that are crucial for dealing with systemic risks become marginalized.

Cox et al. (2022, this issue) focus their contribution on the topic of ripple effects using data from a series of deliberative workshops with UK lay publics on novel methods for removing carbon dioxide from the atmosphere. Using secondary data analysis, they argue that heightened risk perceptions relating to the UK controversy over fracking for unconventional gas have extended via “ripple effects” across technologies, to negatively impact people's perceptions of CO_2_ removal technologies—novel technologies to directly reduce carbon from the atmosphere and thereby potentially help in combating climate change. Such Stage II ripple effects across technologies are an important feature of the original risk amplification papers, but have garnered less attention or empirical verification since. In the workshops, participants’ attitudes were underpinned by deeper misgivings regarding the actions and motives of experts and policymakers; a pervasive discourse of “but they told us it was safe” regarding fracking negatively affected people's trust in assurances of the safety and efficacy of CO_2_ removal. The authors argue that this has the potential to undermine attempts to build societal agreement around future deployment of CO_2_ removal technologies.

## 5 CONTINUING RISK ANALYSIS CONUNDRUMS

SARF was created over three decades ago; therefore, it is not surprising that the risk analysis field and social change have seen a number of new insights and puzzling issues that have arisen. Few areas of research and scholarship stand still. And so the social amplification framework, drawing upon the knowledge and state of the field in the 1980s, has both new possibilities and new challenges that did not exist at the time of writing. Some new issues have called into question the current structure and assumptions of the framework. Others provide opportunities for revising and enlarging the framework—ultimately, the social sciences involve evolution and change and so the framework needs to evolve and become even more robust. Accordingly, we identify a number of these new changes and how analytic and integrated thinking needs to address such changes in a new world of risk assessments. Here we address issues that continue to bedevil risk analysis and for which the social amplification framework may provide some useful gains upon which future analyses may build.

Part of this conversation relates to whether SARF is limited as a framework and a metaphor and is not a theory. Some might argue that this gives utility to the framework for helping to identify hypotheses around opaque or uncertain public views and values that can then be tested—an exploratory tool. Others state that SARF can only be useful as a post hoc approach and is not able to test hypotheses or to be a predictive tool. Do global digital communications and social media channels —not considered in the original framework—make SARF outdated? On the other hand, SARF seems to fit the dynamics of the twitter verse and some researchers have applied the framework to investigate how these communication mechanisms amplify risk. We explore this further in the section below along with other conundrums related to uncertainties, risk communication, integrated analysis, and systemic risks.

### 5.1 Social media

Written in the 1980s, SARF appropriately focused on the role of the mass media in articulating risk signals to the publics and the media articulators as well as conveying signals from risk analysts and decisionmakers. Mass media, initially limited to local and national print and broadcast news, was the primary channel that delivered risk information to the publics and as well as from risk managers. These dynamics shaped people's risk perceptions (Friedman & Sutton, 2020). Over the past two decades, information delivered over social media channels has increasingly affected the way that individuals and institutions interact with one another, directly influencing the patterns of amplification of risk via online networked communication platforms. Van Dijck and Poell (2013) describe a new “social media logic,” characterizing social media as it has contributed to new norms, strategies, mechanisms, and economies, that underpin how and why information is shared online.

For example, social media platforms are supported by sociotechnological architectures that facilitate specific forms of user practices. Consider, for example, how technological affordances allow personal broadcasting that blurs the traditional boundary between mass media and interpersonal communication where the receiver becomes the source of communication (see Sundar, 2008). Or, the user‐generated classification system, known as “folksonomy,” or social tagging (such as hashtags), that makes information easily found by others (Pink, 2005). And, consider the social media functions that facilitate user engagement, such as likes, favorites, comments, retweets, and quote tweets. These features allow organizations and commodified individuals to measure the electronic reach, or amplification, of their digital material and quantify their time and investment (Olsen et al., 2019). They also stimulate algorithms that promote some material to the top of a queue while limiting the visibility of others in a form of algorithmic bias (Brown, 2021). “Echo chambers” are an intrinsic element that emerge as algorithms and serve up content that aligns with the information preferences of individuals. The networked nature of social media further limits exposure to alternate perspectives outside of the closed groups. Since social media channels are highly targeted by algorithms that continue to push similar preferred content, eliciting contrary views may not be possible or even tolerated. While one might argue that the role of mass media previously had been to provide a “balanced” and “objective” perspective, social media channels do not make requirements for truth or malice of those who publish their thoughts online. Clearly, SARF and risk communication are facing a new challenge. When the amplification of content is restricted or promoted as a result of computational decisions determined by the platform engineer, one cannot help but consider that the *social* amplification of risk is far less social on social media.

The socio‐technical architectures of social media also make the content of communication, that is the messages themselves, “nonconsensual” in the sense that no global consensus regarding truth, values of empirical claims, behavioral or cultural norms, etc., can be enforced. This also means that there is value in misinformation; platforms profit from promoting topics that create “moral outrage” (Crockett, 2017), resulting in a type of emotional contagion met with increased online engagement and sharing. Here, the mass media adage “if it bleeds, it leads” finds its reflection online; where stories that inspire fear, disgust, and surprise are more likely to be shared than those that do not (Vosoughi et al., 2018).

How might SARF account for the online ecology, affordances, and algorithms that promote social behaviors online and off that are a direct result of the socio‐technical design of social media communication platforms? One might claim that social media logic dictates the interactions, networks of communication, and prioritization of content resulting in a type of technological determinism. If this is so, is the amplification of risk information online truly social? Future SARF research will benefit from research by those who grapple with the complexities inherent in social media structures. Drawing from scholarship on social networks, diffusion, and contagion is likely to lead to greater insight about the amplification of risk than descriptive case studies that detail the changes in volume and attention over time. Researchers have spent a great deal of effort learning *what* content is amplified when important remaining questions are *why and how* does this influence decision making.

Risk communicators who work to identify those risks that really matter and develop strategies to address what should be done about them, now face new challenges with the advent of a more diffuse social media. Such platforms can enhance the capability to convey needed information, facilitating participation, and allowing multiple voices to contribute and share, or it can become a vehicle for promoting biased, untruthful, or inaccurate views of reality. There is also opportunity, of course. Given that society has changed dramatically, how do we best adapt, and maybe even exploit, this technological transformation in SARF and in risk communication more generally**?** Risk analysis has work to do.

### 5.2 Uncertainty

Uncertainty is inescapable in risk and policy analysis, even in familiar situations—such as crossing a street or driving a car. People rely on existing knowledge and experience to guide future expectations, as Kahneman (2011) has pointed out. But contexts change, and new elements affecting risk unexpectedly appear. For highly complex systems with extensive connectivity and interactions, or novel problems or technology for which experience provides little guidance, decisions must often be made quickly and under conditions of high uncertainty, greatly complicating the assessment of risk.

Uncertainty arises from gaps in data, insufficient models, or incomplete scientific understanding of a risk. Indeed, we recognize these types of uncertainty (statistical, model parameters, and epistemological). Depending on the type and source of uncertainty, new information and more data may not reduce uncertainty. As was noted in *Thinking Strategically*, a National Research Council report (NRC, 2005), scientific progress may not only reduce some uncertainties but also uncover new ones.

It is not surprising that—in a world of complex systems involving rapid technological change, highly coupled human and natural systems, and a kaleidoscope of social, economic, and political institutions—high levels of uncertainty challenge existing assessment methods as well as public consideration and communication of risk decision and management procedures (Goble et al., 2017). In *Science and Decisions: Advancing Risk Assessment* (NRC, 2009), a committee of experts identified core principles for addressing uncertainty and variability in risk assessments.

Uncertainty is as much a management problem as an assessment problem. Much of the literature on uncertainty presumes that, if we just characterize uncertainty properly, managers will know what to do. But in fact, managers are apt to be baffled by and resist ever more those “proper” characterizations of uncertainty. Risk managers need guidance about what to do and what not to do when the assessments do not point to a clear path forward (Goble et al., 2017). Certainly, risk managers need to communicate about risk, and it is here that SARF offers insights because it is obvious that uncertainty can strongly influence social amplification processes.

SARF offers several contributions to more effective uncertainty analysis. First, since hazards and risk are defined more broadly, a more diverse set of risks are identified. And so, the uncertainties may be broader and more complex. Inevitably, a greater number of uncertainties are involved. Yet, even more challenging, hazards to what people value and how values come into play are inevitably more perplexing. And so the uncertainties are more numerous and greater for the elements of risk that are more poorly understood. For managers and analysts, the challenges are there. Ordinary people and decisionmakers dislike uncertainty—the judgment can be made that the problems are really not understood. And so, the analyst is confronted with the inextricable challenge—what uncertainties should be communicated and what do we do about poorly understood risks?

### 5.3 Risk communication

Risk communication is a third continuing problem for risk analysis. The past 30 years has seen a flood of work on risk communication initiatives and analyses (Árvai & Rivers, 2014; Rickard, 2021). The earlier works of NRC (1983, 1989), Granger Morgan (see, for example, Morgan & Lave, 1990), Fischhoff (1995), and Chess et al. (1995) are noteworthy contributions that should not be lost in the ongoing publications on risk communication. And yet the practice of risk communication by corporations, federal agencies, and ideal government in many respects seem little changed alas, from practice decades ago—albeit social media has changed the methods drastically. The time is overdue to address some tough questions for the architects and craftsmen who shape and implement the practice of risk communication. Social amplification thinking can contribute. A retrospective look at risk communication proceeds with four major questions (Kasperson, 2014)^2^:

- What major successes and failures can we point to that shed light on what has been learned and not learned since the 1983 NRC report?
- Assessing and communicating uncertainty often befuddles decisionmakers and risk managers. How are these needs handled, and how well, in current practice and analysis? How can we do better?
- While risks are an inescapable part of the governance and democratic process the reservoir of social trust is and has been in long‐term decline. How successfully is declining trust handled in risk governance processes?
- Can the lessons learned and answers to the above be translated into a new list of principles for risk communication going forward?

### 5.4 Integrated analysis

In its early stages, risk analysis was essentially a technical activity. In the 1970s, a contribution shaped the objectives and structure of risk analysis for nuclear accidents and eventually contributed to the creation of the “Red Book” (NRC, 1983), which became the bible of governmental agencies and, in turn, large corporations. Commissioned by the first Administrator of the EPA, William Ruckelshaus, to guide EPA's decision making about toxic chemicals and contaminated sites, the report was influenced, indirectly, by nuclear risk assessments. At the same time, however, studies of the social issues involved in risk were growing rapidly, particularly psychometric and cultural studies of risk perception among lay publics by Slovic, Fischhoff, and others. And yet, these domains of analysis remained largely unintegrated and the “Red Book” remains the primary document in federal and state governments for how risk issues should be addressed. SARF survives as the most promising analytic strategy to achieve integrated analysis in which technical and social risk are examined in their interaction. Interdisciplinary approaches must draw upon psychology, sociology, political science, cultural theories, and economics and this requires integration of different vocabularies, literatures, and epistemologies (Neeley, 2014).

### 5.5 Systemic risk

Risk analysis has suffered from a propensity to analyze issues one by one for specific technologies, facilities, or places. A major step to a more integrated analysis is to see issues in their systems profile, not only their local or place‐based perspectives. And yet, if risk analysis is viewed over time, it is quite apparent that the bulk of the risk effort has focused on individual chemicals, facilities, technologies, or places.

SARF made perhaps the earliest attempt to describe the idea of systemic risk. It introduced the idea of risk ripples as secondary and tertiary consequences of a hazard event that dominoed through interconnected systems. It also explained that these ripples were carried through human connections as ideas about hazards and risks were communicated and shaped into perceptions and behaviors (see Larson et al., 2022, this issue).

Renn and Klinke (2016) have proposed strategies for getting out of this mold. Renn, also a major author of the social amplification framework, has focused on the larger issue of risk governance and, to follow, upon the systems properties that enter into governance, writ broadly, and in decision making on diverse risk problems. And so they have recognized the need for integrated analysis of systems, risks, and regions. Accordingly, they argue that, for risk management to be effective, it must proceed through the stages: preestimation, interdisciplinary risk estimation (including risk assessment and concern assessment, integrated risk analysis, and risk management.

The governance framework will be debated, of course, but the need for more integrated and systemic perspectives are recognized and set forth. The intent is to engage three principal problems in risk analysis; complexity, uncertainty, and ambiguity. Systemic risk, in Renn's (2017) view, seeks four major objectives: be global in nature, be interconnected and intertwined, nonlinear in cause and effect, and be stochastic in effect structure. These are steps that need to be considered for how best the risk analysis field can move forward.

## 6 CONCLUSION: PRIORITIES FOR MOVING FORWARD

SARF is both a metaphor to refute one dimensional technical risk and an architecture to facilitate the linking of cultural, social, and psychological theories of risk. The driving questions that remain are: why and how do some risks undergo amplification or attenuation? While not a predictive theory of social outcomes, SARF has been demonstrated to be a very effective tool for exploring hypotheses around how risk perceptions form and it has played important roles in informing risk communication and collaborative risk governance between regulators, experts, and laypeople.

As one of the most cited articles in *Risk Analysis*, SARF has stood the test of time, yet there are considerable opportunities for development. Notably among those are the importance of social media (and multiple feedbacks) in shaping risk perceptions and the psychometric work focused on the stigma that may develop for particular places. SARF could become more effective if it was able to link to a ledger of more social and behavioral theories of risk (e.g., affect heuristic, tragedy of the commons).

Social and generalized trust receive a polite nod in most risk analysis work and not much more. Analysts have shown that trust is complex and multidimensional. What are the implications for proceeding with risk management when trust is low or high? Is there a “trust deficit” and what role would it play in the amplification of risk? Should risk communication and governance proceed differently if trust is in short supply or if it exists in abundance? Also needed is in‐depth research in different cultural settings with varied governance structures. Perhaps a new framework or a major revision of SARF is needed for how amplification and attenuation occur in different contextual and social settings. More study is also needed on the various types of signals and related ripple effects, particularly around the attenuation of risk perceptions. And we need additional practical applications of SARF that can help in the management and governance of risks.

SARF has evolved into an effective organizational aide for examining different types of qualitative information sources around historical public responses. Its utility remains focused on how risk language is communicated across social stations, including the role of information and misinformation, and how this translates to changing risk perceptions and impacts of the risk on communities.

## Footnotes

1 The original SARF analysis applied the case study of the 1989 explosion at the chemical plant in Bhopal, India that raised concerns about the possible failure of “fail‐safe” systems at nuclear power plants (Kasperson & Kasperson 1991).

2 For several responses to Kasperson's four major questions, see the *Journal of Risk Research*, (17)10, pp. 1241–1284.

## ARTICLES ABOUT SARF SINCE 2003

### 1

Adekola, J. (2019). A critique of the social amplification of risk framework from the power perspective. *Power and risk in policy making: Understanding public health debates* (pp. 27–41), Cham Switzerland: Palgrave Pivot.

Adekola, J. (2019). The Role of Power and Expertise in Social Amplification of Risk. *Power and risk in policy making: Understanding public health debates* (pp. 103–118). Cham Switzerland: Palgrave Pivot.

Adekola, J., Fischbacher‐Smith, D. and Fischbacher‐Smith, M. (2019) Light me up: power and expertise in risk communication and policy‐making in the e‐cigarette health debates. *Journal of Risk Research*, *22*(10), pp. 1294–1308. (doi: https://doi.org/10.1080/13669877.2018.1473463)

Anyfantis, I., & Karageorgiou, A. (2020). The social amplification and attenuation of risk on occupational safety and health leveling‐the case of Greece. *International Journal of Occupational and Environmental Safety*, *4*(2), 12–21.

Arvai, J., & Rivers, L. (Eds.). (2014). Effective risk communication. London, UK: Routledge.

Aula, Pekka. (2010). Social Media, Reputation Risk and Ambient Publicity Management. *Strategy & Leadership, 38*(6), 43–49.

Bakir, V. (2005). Greenpeace v. Shell: Media exploitation and the social amplification of risk framework (SARF). *Journal of Risk Research*, *8*(7–8), 679–691.

Barnett, J., & Breakwell, G. M. (2003). The social amplification of risk and the hazard sequence: the October 1995 oral contraceptive pill scare. *Health, risk & society*, *5*(3), 301–313.

Bennett, S. (2005). The role of social amplification and news values in the re‐presentation of risk research: A case study. *Risk Management*, *7*(1), 9–29.

Binder, A. R., Scheufele, D. A., Brossard, D., & Gunther, A. C. (2011). Interpersonal amplification of risk? Citizen discussions and their impact on perceptions of risks and benefits of a biological research facility. *Risk Analysis*, *31*(2), 324–334.

Binder, A. R., Cacciatore, M. A., Scheufele, D. A., & Brossard, D. (2014). The role of news media in the social amplification of risk. In H. Cho & T. Reimer (Eds.), *The SAGE handbook of risk communication* (vol. 69), Thousand Oaks, CA: Sage.

Brenkert‐Smith, H., Dickinson, K. L., Champ, P. A., & Flores, N. (2013). Social amplification of wildfire risk: the role of social interactions and information sources. *Risk Analysis*, *33*(5), 800–817.

Busby, J. S., Alcock, R. E., & MacGillivray, B. H. (2009). Interrupting the social amplification of risk process: a case study in collective emissions reduction. *Environmental Science & Policy*, *12*(3), 297–308.

Busby, J., & Duckett, D. (2012). Social risk amplification as an attribution: The case of zoonotic disease outbreaks. *Journal of Risk Research*, *15*(9), 1049–1074.

Busby, J. S., & Onggo, S. (2013). Managing the social amplification of risk: a simulation of interacting actors. *Journal of the Operational Research Society*, *64*(5), 638–653.

Cantrill, J. G. (2011). The role of a sense of self‐in‐place and risk amplification in promoting the conservation of wildlife. *Human Dimensions of Wildlife*, *16*(2), 73–86.

Carper, L. B. (2019). What influences our decision to vaccinate? The social amplification of risk framework. *Proceedings of the International Crisis and Risk Communication Conference, Volume 2* (pp. 12–14). Orlando, FL: Nicholson School of Communication and Media. https://doi.org/10.30658/icrcc.2019.3

Chong, M., & Choy, M. (2018). The social amplification of haze‐related risks on the internet. *Health Communication*, *33*(1), 14–21.

Chung, I. J. (2011). Social amplification of risk in the Internet environment. *Risk Analysis*, *31*(12), 1883–1896.

Chung, J. B., & Yun, G. W. (2013). Media and social amplification of risk: BSE and H1N1 cases in South Korea. *Disaster Prevention and Management: An International Journal*, *22*(2):148–159.

Comrie, E. L., Burns, C., Coulson, A. B., Quigley, J., & Quigley, K. F. (2019). Rationalizing the use of Twitter by official organisations during risk events: Operationalising the social amplification of risk framework through causal loop diagrams. *European Journal of Operational Research*, *272*(2), 792–801.

Crespi, I., & Taibi, M. (2020). Cultural Differences and Social Amplification of Risk of a Tourism Destination: Foreign Media Coverage after 2016/2017 Earthquakes in Central Italy. *Italian Sociological Review*, *10*(2), 201–237.

Duckett, D., & Busby, J. (2013). Risk amplification as social attribution. *Risk Management*, *15*(2), 132–153.

Fan, Y. (2020, October). The social amplification of risk on Weibo‐Take the COVID‐19 epidemic in China as an example. In *Proceedings of the 2020 Conference on Artificial Intelligence and Healthcare* (pp. 91–95).

Fellenor, J, Barnett, J., Potter, C., Urquhart, J., Mumford J. D. & Quine C. P. (2018) The social amplification of risk on Twitter: the case of ash dieback disease in the United Kingdom, *Journal of Risk Research, 21*(10), 1163–1183, DOI: 10.1080/13669877.2017.1281339

Fellenor, J., Barnett, J., Potter, C., Urquhart, J., Mumford, J. D., & Quine, C. P. (2020). ‘Real without being concrete’: the ontology of public concern and its significance for the Social Amplification of Risk Framework (SARF). *Journal of Risk Research*, *23*(1), 20–34.

Fjaeran, L., & Aven, T. (2021). Making visible the less visible–how the use of an uncertainty‐based risk perspective affects risk attenuation and risk amplification. *Journal of Risk Research, 24*(6), 673–691.

Hart, P. S., Nisbet, E. C., & Shanahan, J. E. (2011). Environmental values and the social amplification of risk: An examination of how environmental values and media use influence predispositions for public engagement in wildlife management decision making. *Society and Natural Resources*, *24*(3), 276–291.

Hopfer, S., Fields, E. J., Lu, Y., Ramakrishnan, G., Grover, T., Bai, Q., … & Mark, G. (2021). The social amplification and attenuation of COVID‐19 risk perception shaping mask wearing behavior: A longitudinal twitter analysis. *PloS one*, *16*(9), e0257428.

Jagiello, R. D., & Hills, T. T. (2018). Bad news has wings: Dread risk mediates social amplification in risk communication. *Risk Analysis*, *38*(10), 2193–2207.

Kandiah, V., Binder, A. R., & Berglund, E. Z. (2017). An empirical agent‐based model to simulate the adoption of water reuse using the social amplification of risk framework. *Risk Analysis*, *37*(10), 2005–2022.

Kao, S. F. (2008). Social amplification of risk and environmental collective activism: a case study of Cobalt‐60 contamination incident in Taiwan. *International Journal of Global Environmental Issues*, *8*(1–2), 182–203.

Kasperson, R. E. (2005). *Social contours of risk: Publics, risk communication and the social amplification of risk*. Washington D. C.: Earthscan.

Kasperson, R. E. (2012). A perspective on the social amplification of risk. *The Bridge*, *42*(3), 23–27.

Kasperson, J. X., Kasperson, R. E., Pidgeon, N., & Slovic, P. (2012). The social amplification of risk: Assessing 15 years of research and theory. In *Social contours of risk* (pp. 217–245). New York: Routledge.

Kasperson, R. E. (2015). Risk governance and the social amplification of risk: A commentary. In *Risk Governance* (pp. 485–487). Springer, Dordrecht.

Kasperson, R., Jhaveri, N., & Kasperson, J. X. (2013). Stigma and the social amplification of risk: Toward a framework of analysis. In *Risk, media and stigma* (pp. 25–44). New York: Routledge.

Kasperson, J. X., Kasperson, R. E., Pidgeon, N., & Slovic, P. (2013). The social amplification of risk: assessing fifteen years of research and theory. In *The feeling of risk* (pp. 345–372). New York: Routledge.

Kim, K. H., Choi, J. W., Lee, E., Cho, Y. M., & Ahn, H. R. (2015). A study on the risk perception of light pollution and the process of social amplification of risk in Korea. *Environmental Science and Pollution Research*, *22*(10), 7612–7621.

Kuentzel, W. F., Daigle, J. J., Chase, L. C., & Brown, T. L. (2018). The social amplification of risk and landowner liability fear in the US Northern Forest. *Journal of Outdoor Recreation and Tourism*, *21*, 51–60.

Lee, Y. C., Park, Y. S., Yun, Y. S., & Kim, J. S. (2015). A Study on the Application and Proposals of Safety Culture, New Public Management and Social Amplification of Risk Framework via Ship Accidents in Korea. *Journal of the Korean Society of Marine Environment & Safety*, *21*(3), 283–289.

Lewis, R. E., & Tyshenko, M. G. (2009). The impact of social amplification and attenuation of risk and the public reaction to mad cow disease in Canada. *Risk Analysis: An International Journal, 29*(5), 714–728. doi: 10.1111/j.1539‐6924.2008.01188.x

Mase, A. S., Cho, H., & Prokopy, L. S. (2015). Enhancing the Social Amplification of Risk Framework (SARF) by exploring trust, the availability heuristic, and agricultural advisors' belief in climate change. *Journal of Environmental Psychology*, *41*, 166–176.

Moussaïd, M., Brighton, H., & Gaissmaier, W. (2015). The amplification of risk in experimental diffusion chains. *Proceedings of the National Academy of Sciences*, *112*(18), 5631–5636.

Niu, C., Jiang, Z., Liu, H., Yang, K., Song, X., & Li, Z. (2020). The influence of media consumption on public risk perception: A meta‐analysis. *Journal of Risk Research, 25*(1), 21–47.

Ng, Y. J., Yang, Z. J., & Vishwanath, A. (2018). To fear or not to fear? Applying the social amplification of risk framework on two environmental health risks in Singapore. *Journal of Risk Research*, *21*(12), 1487–1501.

Onggo, B. S. (2017). Social amplification of risk framework: An agent‐based approach. In *Advances in Social Simulation 2015*, (pp. 335–339). Cham, Switzerland: Springer.

Park, S., & Smardon, R. C. (2011). Worldview and social amplification of risk framework: dioxin risk case in Korea. *International Journal Applied Environmental Science 6*(2), 173–191.

Pidgeon, Nick, and Julie Barnett. (2013). Chalara and the Social Amplification of Risk. London: DEFRA. https://www.gov.uk/government/uploads/system/uploads/attachment_data/file/200394/pb13909–chalara‐social‐amplification‐risk.pdf

Popovic, N. F., Bentele, U. U., Pruessner, J. C., Moussaïd, M., & Gaissmaier, W. (2020). Acute stress reduces the social amplification of risk perception. *Scientific reports*, *10*(1), 1–11.

Renn, O. (2011). The social amplification/attenuation of risk framework: Application to climate change. *Wiley Interdisciplinary Reviews: Climate Change*, *2*(2), 154–169.

Rickard, L. N., McComas, K. A., Clarke, C. E., Stedman, R. C., & Decker, D. J. (2013). Exploring risk attenuation and crisis communication after a plague death in Grand Canyon. *Journal of Risk Research*, *16*(2), 145–167.

Sacilotto, J., & Loosemore, M. (2018). Chinese investment in the Australian construction industry: the social amplification of risk. *Construction Management and Economics*, *36*(9), 507–520.

Sato, H., & Webster, A. (2022). Mixed effects of mass media reports on the social amplification of risk: frequencies and frames of the BSE reports in newspaper media in the UK. *Journal of Risk Research, 25*(1), 48–66.

Schneider, E. J., Boman, C. D., & Akin, H. (2021). The Amplified Crisis: Assessing Negative Social Amplification and Source of a Crisis Response. *Communication Reports*, *34*(3), 165–178.

Shakeela, A., & Becken, S. (2015). Understanding tourism leaders’ perceptions of risks from climate change: An assessment of policy‐making processes in the Maldives using the social amplification of risk framework (SARF). *Journal of Sustainable Tourism*, *23*(1), 65–84.

Stanciugelu, I., Frunzaru, V., Armas, I., Duntzer, A., & Stan, S. (2013). Social amplification of risk and crisis communication planning‐a case study. *Scientific Bulletin‐Nicolae Balcescu Land Forces Academy*, *18*(2), 197–205.

Strekalova, Y. A. (2016). Health risk information engagement and amplification on social media: News about an emerging pandemic on Facebook. *Health Education & Behavior*, *44*(2), 332–339.

Strekalova, Y. A., & Krieger, J. L. (2017). Beyond words: amplification of cancer risk communication on social media. *Journal of Health Communication*, *22*(10), 849–857.

Urquhart, J., Potter, C., Barnett, J., Fellenor, J., Mumford, J., & Quine, C. P. (2017). Expert risk perceptions and the social amplification of risk: A case study in invasive tree pests and diseases. *Environmental Science & Policy*, *77*, 172–178.

van Goudoever, M. J., Mulderij‐Jansen, V. I., Duits, A. J., Tami, A., Gerstenbluth, I. I., & Bailey, A. (2021). The Impact of Health Risk Communication: A Study on the Dengue, Chikungunya, and Zika Epidemics in Curaçao, Analyzed by the Social Amplification of Risk Framework (SARF). *Qualitative Health Research, 31*(10), 1801–1811.

Vijaykumar, S., Jin, Y., & Nowak, G. (2015). Social media and the virality of risk: The risk amplification through media spread (RAMS) model. *Journal of Homeland Security and Emergency Management*, *12*(3), 653–677.

Vulpe, S. N., & Rughiniş, C. (2021). Social amplification of risk and “probable vaccine damage”: A typology of vaccination beliefs in 28 European countries. *Vaccine*, *39*(10), 1508–1515.

Wardman, J. K., & Löfstedt, R. (2018). Anticipating or accommodating to public concern? Risk amplification and the politics of precaution reexamined. *Risk Analysis*, *38*(9), 1802–1819.

Wirz, C. D., Xenos, M. A., Brossard, D., Scheufele, D., Chung, J. H., & Massarani, L. (2018). Rethinking social amplification of risk: Social media in Zika in three languages. *Risk Analysis, 38*(12), 2599–2624. https://doi.org/10.1111/risa.13228

Zhang, L., Xu, L., & Zhang, W. (2017). Social media as amplification station: Factors that influence the speed of online public response to health emergencies. *Asian Journal of Communication*, *27*(3), 322–338.

Zhang, X. A., & Cozma, R. (2022). Risk sharing on Twitter: Social amplification and attenuation of risk in the early stages of the COVID‐19 pandemic. *Computers in Human Behavior*, *126*, 106983.

Zhao, S., & Wu, X. (2021). From Information Exposure to Protective Behaviors: Investigating the Underlying Mechanism in COVID‐19 Outbreak Using Social Amplification Theory and Extended Parallel Process Model. *Frontiers in Psychology*, *12*, 1351.
